# Pluripotency Genes and Their Functions in the Normal and Aberrant Breast and Brain

**DOI:** 10.3390/ijms161126024

**Published:** 2015-11-13

**Authors:** Tracy Seymour, Alecia-Jane Twigger, Foteini Kakulas

**Affiliations:** 1 School of Chemistry and Biochemistry, Faculty of Science, the University of Western Australia, Perth, Western Australia 6009, Australia; tracy.seymour@research.uwa.edu.au (T.S.); alecia.twigger@uwa.edu.au (A.-J.T.); 2 School of Medicine and Pharmacology, Faculty of Medicine, Dentistry and Health Sciences, the University of Western Australia, Perth, Western Australia 6009, Australia

**Keywords:** pluripotency genes, oncogenes, OCT4, SOX2, NANOG, pluripotent stem cells, embryonic stem cells, adult stem cells, cancer stem cells, breast, mammary gland, brain, breast cancer, brain cancer, cancer

## Abstract

Pluripotent stem cells (PSCs) attracted considerable interest with the successful isolation of embryonic stem cells (ESCs) from the inner cell mass of murine, primate and human embryos. Whilst it was initially thought that the only PSCs were ESCs, in more recent years cells with similar properties have been isolated from organs of the adult, including the breast and brain. Adult PSCs in these organs have been suggested to be remnants of embryonic development that facilitate normal tissue homeostasis during repair and regeneration. They share certain characteristics with ESCs, such as an inherent capacity to self-renew and differentiate into cells of the three germ layers, properties that are regulated by master pluripotency transcription factors (TFs) OCT4 (octamer-binding transcription factor 4), SOX2 (sex determining region Y-box 2), and homeobox protein NANOG. Aberrant expression of these TFs can be oncogenic resulting in heterogeneous tumours fueled by cancer stem cells (CSC), which are resistant to conventional treatments and are associated with tumour recurrence post-treatment. Further to enriching our understanding of the role of pluripotency TFs in normal tissue function, research now aims to develop optimized isolation and propagation methods for normal adult PSCs and CSCs for the purposes of regenerative medicine, developmental biology, and disease modeling aimed at targeted personalised cancer therapies.

## 1. Introduction

In normal development, embryonic stem cells (ESCs) drive embryogenesis and differentiate into the three germ layers of ectoderm, endoderm and mesoderm, which generate the complete organism [1]. Self-renewal (cell proliferation) rate, differentiation capability, karyotype integrity, telomere length and telomerase activity are all maintained in ESCs, even after multiple passages, establishing the pluripotent state, which is conserved *in vitro* [2,3]. *In vivo* studies of pluripotency in murine ESCs include evaluating chimera integration and teratoma formation after injection, however only the latter is used to investigate human ESCs due to ethical reasons [4]. Gene expression is also a major consideration when investigating ESCs, with OCT4 (octamer-binding transcription factor 4), SOX2 (sex determining region Y-box 2), and homeobox protein NANOG being recognized as master transcription factors (TFs) controlling pluripotency [5] and thus, the early stages of embryogenesis. Interestingly, in more recent years, pluripotent stem cell (PSC) properties have been described for certain cell populations outside the embryonic stage, in the adult organism [6]. Pluripotency genes, influencing numerous downstream targets, are tightly regulated both in the embryo and in the adult to orchestrate normal development and function, and when deregulated, they have been associated with pathologies such as cancer [7,8]. Here, we discuss the importance of pluripotency genes during embryogenesis, emphasizing that they are also vital components of normal self-renewal and differentiation capacities in certain types of adult stem cells, such as in the breast and brain. We further present the recently reported role of pluripotency genes in mediating normal mammary development during pregnancy and lactation [9], and normal cell turnover in the neural system [10,11]. We then explore the malignant effects of deregulation of pluripotency TFs acting as oncogenes in these organs, implicating the use of technologies that specifically target pluripotency oncogenes as novel cancer therapies.

## 2. Pluripotency Genes and Their Role in Embryogenesis

TFs OCT4, SOX2 and NANOG are considered the master regulators of pluripotency in ESCs due to their ability to activate downstream targets that regulate self-renewal and differentiation [5,12]. *OCT4*, a member of the Pit-Oct-Unc (POU) TF family, was the first gene noted to be essential for the successful formation of pluripotent inner cell mass cells in the blastocyst during embryogenesis [13]. Later, *SOX2*, encoding a highly conserved high mobility group (HMG) DNA binding domain [5], was found to heterodimerize via protein-protein interactions with OCT4 to synergistically activate and repress several genes associated with self-renewal and differentiation. NANOG, a homeobox protein, is a known downstream target of OCT4 and SOX2, and together the three genes are thought to be the central regulators of several other genes that balance self-renewal and differentiation in ESCs [5,14]. In addition to *SOX2*, *OCT4* and *NANOG*, other genes such as *KLF4*, *REX1*, *SSEA3*, *SSEA4*, *TRA-1-60* and *TRA-1-81* are involved in and co-regulate the complex pluripotency circuitry in ESCs [5,15,16].

**Figure 1:**
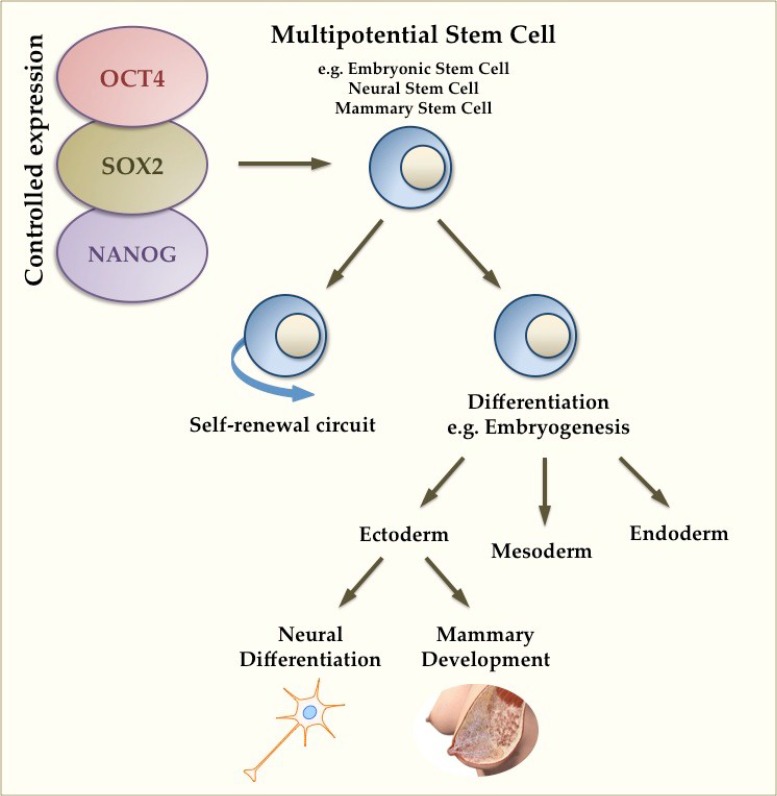
Controlled expression of pluripotency genes in multipotential stem cells. Tight regulation of pluripotency genes *OCT4*, *SOX2* and *NANOG* controls the balance between self-renewal and differentiation. Multipotential stem cells are present during embryonic stages and control the formation of the three germ layers: ectoderm, mesoderm and endoderm. The mammary gland picture is reproduced with permission from Medela AG.

OCT4, SOX2 and NANOG are pivotal to our understanding and characterization of ESCs and other PSCs, playing key roles in controlling lineage-specific differentiation required for the formation of cells from the three germ layers (ectoderm, endoderm and mesoderm) [5] (Figure 1). OCT4 promotes cells towards the mesodermal lineage, suppresses ectodermal lineage differentiation, and is downregulated along with NANOG during endodermal differentiation [2,17]. On the other hand, SOX2 suppresses mesodermal differentiation and is upregulated in clonally derived human embryonic cell lines at ectodermal and neural tube formation during neuroectodermal differentiation [2] (Figure 1). Interestingly, *NANOG* expression is thought to be restricted to PSCs and is downregulated in an exponential fashion during differentiation and embryonic development [2,18]. Additionally, these TFs control the transcriptional regulation of their own promoter genes creating an autoregulatory loop [5]. This demonstrates a mechanism in which stem cell identity is maintained whilst still allowing for the influence of cell fate cues [5,18]. The autoregulation of *OCT4*, *SOX2* and *NANOG* is highly conserved, emphasising its importance in normal stem cell function [5].

## 3. Pluripotency Genes in Adult Stem Cells

The bone marrow is the most widely studied stem cell niche in the adult, however many other tissues and fluids such as the dental pulp, cord blood, breastmilk, the basement membrane of the seminiferous tubules, and the endometrium contain stem cells with pluripotent features [19,20,21,22,23]. Mesenchymal/stromal stem cells (MSCs) from the bone marrow are defined by their ability to differentiate into osteoblasts, adipocytes and chondrocytes and express specific markers including CD44, CD63, CD105 and CD146 [24]. By this definition, MSCs can be identified in a range of other adult human tissues and fluids, such as peripheral blood, umbilical cord blood, adipose tissue, saliva and the dental pulp [25,26,27,28,29]. There, subpopulations of cells with pluripotent characteristics have also been described. These include the dental pulp pluripotent-like stem cells (DPPSCs), which express the core pluripotency TFs, proliferate with similar morphology to hESCs, form multilineage teratomas in immunodeficient mice, and create functional neurons [30,31]. Similarly, umbilical cord blood cells have many pluripotent features, including extensive proliferation capacity in culture, the ability to differentiate into the classical mesenchymal lineages, but also into neural, hepatic and cardiac cells, and longer telomere length than MSCs [32]. Most recently, pluripotent-like cells have been isolated from the human minor salivary gland and have been termed human minor salivary gland mesenchymal stem cells (hMSGMSCs) [33]. *In vitro* studies showed that hMSGMSCs maintain stem cell features, demonstrate high expression of CD29, CD44 and CD73, and differentiate in culture towards the mesodermal lineage after direct induction. *In vivo*, hMSGMSCs did not form teratomas, but were able to survive and proliferate when injected into damaged liver tissue, implicating their potential use in regenerative medicine [33].

Interestingly, scarce subpopulations of stem cells within the bone marrow have also been shown to harbour pluripotency features. Murine bone marrow contains cells termed very small embryonic-like (VSEL) stem cells that express pluripotency markers such as OCT4, SOX2, NANOG, SSEA4 and REX1, and have multilineage differentiation capabilities, being able to differentiate *in vivo* into retinal neurons and insulin-producing cells [34,35,36,37,38]. Similarly, multilineage differentiating stress enduring (MUSE) stem cells from the human bone marrow display features of pluripotency, and differentiate and integrate *in vivo* within damaged sites of immunodeficient mice, yet they do not form teratomas [39]. In addition, a scarce population of tiny stem cells termed StemBios (SB), smaller than 6 μm in diameter, has been recently identified in the bone marrow with multilineage potential *in vitro* and *in vivo*, expressing some but not all of the classical pluripotency markers [23].

Some reproductive organs, such as the testes and the endometrium, also contain pluripotent-like cells, which express pluripotency genes [19,22]. These cells have been proposed as stem cell candidates for regenerative medicine, but require dedifferentiating in defined media or slight retroviral transduction to generate enhanced pluripotent cells. Another reproductive organ, the breast, naturally contains pluripotent-like cells that can be non-invasively accessed via breastmilk [19,22,40,41].

In addition to cells that satisfy the two main criteria of pluripotency (self-renewal and differentiation into the three germ layers), cells with partial pluripotency features, such as expression of certain but not all pluripotency TFs, and with more limited differentiation capacities, have been described in both reproductive organs and other tissues, such as the heart, liver, pancreas and brain [42,43]. The purpose(s) of these specialised cells are still unclear. It has been hypothesised that they remain quiescent in adult tissues and are activated by the onset of tissue damage for the purposes of tissue repair and regeneration. Also, epigenetic changes during development may reduce their tumourigenicity preventing them from forming teratomas in immunodeficient mice. Therefore, these cells may be remnants of embryonic development and serve important organ regeneration, repair and remodelling functions during adult life [38,40,41].

### 3.1. Pluripotency Genes in Normal Breast Development

The mammary gland is the only organ in the body that fully matures in adult life during pregnancy and lactation, and contains cells with pluripotent features at different stages of its development [20]. The breast is produced from mammary epithelial buds derived from the ectodermal germ layer (Figure 1), and undergoes very little development until puberty, where a mini pregnancy-like surge of hormones induces stromal and epithelial development of the gland [44]. However, it is not until pregnancy that the mammary tissue progressively matures, with massive remodelling occurring that involves extensive ductal branching, secretory differentiation and alveolar morphogenesis facilitated by the lactogenic hormonal circuit (estrogen, progesterone and prolactin) [20,45,46,47]. After birth, secretory activation in the lactocytes upon decrease in circulating progesterone levels stimulates copious milk synthesis [20,47].

The breast contains heterogeneous mammary stem cells that fuel this massive remodelling via a cellular hierarchical differentiation, with the different cell stages being present throughout lactation [9,40,48]. This cellular hierarchy includes early-stage stem cells that display pluripotency features, mammary committed progenitor cells (luminal and basal), and more differentiated lactocytes (milk-secretory cells) and myoepithelial cells (facilitating alveolar contraction and milk flow towards the nipple) [40,49,50]. Although pluripotent-like stem cells are present in large numbers in the lactating gland, they have also been found as rare subpopulations of cells with both *in vitro* and *in vivo* pluripotency features in the normal resting breast (from non-pregnant, non-lactating women) [40,49,51]. These cells from the resting breast have been shown to display many features of pluripotency, including expression of pluripotency TFs (OCT4, SOX2, NANOG), teratoma formation, and tri-germ layer differentiation capability, yet they are mortal cells with extensive but finite self-renewal [49].

In contrast, the respective cells non-invasively isolated from the human lactating breast via breastmilk have not been shown to form teratomas when injected subcutaneously in immunodeficient SCID mice, rendering them non-tumourigenic [20,21,23,40,45,48]. In turn, these lactation-associated cells, which have been termed breastmilk stem cells (BSCs), have self-renewal and multilineage capabilities *in vitro*, and they have been shown to survive and cross the gastrointestinal tract mucosa of nursed mouse pups *in vivo*, transfer into the bloodstream and from there to different organs where they integrate and differentiate into functional cells [40,52]. These experiments provide evidence supporting both the *in vitro* and *in vivo* pluripotency of BSCs in the right microenvironment, and highlight their non-tumourigenicity. Interestingly, embryonic TFs OCT4, SOX2, NANOG and KLF4 have been detected in not only female, but also male resting mammary tissue [53]. During pregnancy and lactation, a significant upregulation of these genes in specific cell populations within the female breast occurs, an event that is potentially hormonally induced, and which is thought to fuel the remodelling of the gland into a milk-secretory organ. The lack of teratoma formation capabilities in these cells, similar to other adult PSCs known to contribute to tissue regeneration *in vivo* [54], has been attributed to epigenetic changes that are aimed at protecting the adult breast from tumourigenesis, whilst maintaining cell properties essential for the remodelling of this organ during pregnancy and lactation [41]. In addition, these cells, which are abundant in breastmilk, may have specific functions in the infant [41,55].

Indeed, further analysis of pluripotency genes in BSCs revealed expression of all major pluripotency regulators (*OCT4*, *SOX2*, *NANOG*, *KLF4*, *REX1*, *GDF3* and *ESRRB*) as well as correlations with maternal and infant characteristics [56]. In particular, SOX2 was associated with the gestational age of the infant at delivery and the change in breast cup size of the mother during pregnancy, giving further insight into the purposes and potential functions of these pluripotent-like cells in the mother’s breast and in breastmilk [56]. Importantly, preterm birth and maternal obesity were both associated with immature development of the mammary epithelium, which considering the known low milk supply of some of these mothers, provides further insight into the role of pluripotency genes in the remodelling of the gland to prepare it for lactation [56]. Interestingly, differing expression statuses for these genes and their downstream targets were found between mothers who had a boy *versus* a girl [56], suggesting that the embryo influences the development of the mammary gland, and proposing a link between embryonic and mammary development that requires further investigation.

### 3.2. Pluripotency Genes in the Normal Adult Brain

The brain is another organ that undergoes remodelling and is derived from the ectodermal lineage, similar to the mammary gland, containing stem cells governed by pluripotency genes. Neural stem cells (NSCs) are self-renewing and have the ability to differentiate into several neural cell types including neurons, astrocytes and oligodendrocytes [46]. They reside in a specialised microenvironment or niche located in the subventricular zone of the lateral ventricle and the subgranular zone in the hippocampal formation [48,57]. The adult NSC niche is fundamental for supporting self-renewal, activation and differentiation of NSCs [58]. Most importantly, many signalling pathways within the NSC niche determine the fate of its residing stem cells. Hedgehog signalling within both the subventricular and subgranular zone is required for the establishment and maintenance of the neural stem cell pool [59,60]. Mitogen signalling, including fibroblast growth factor, epidermal growth factor and vascular endothelial growth factor, is involved in cell proliferation during neurogenesis [61,62,63]. Wnt signalling appears to induce neuron differentiation, whereas Notch signalling in the subventricular zone prevents neural differentiation and migration [64,65]. Further, newly created neurons demonstrate the ability to migrate and incorporate into pre-existing neuronal areas, retaining normal brain function [66].

In addition to microenvironmental signalling from the NSC niche, NSC properties are maintained through expression of pluripotency genes. *SOX2* is a major player controlling NSC self-renewal and differentiation into neurons or astrocytes [10,11,63]. Within the subventricular zone, cells that express *SOX2* and co-express the glial marker *GFAP* and stem cell marker nestin are thought to function as neurogenic stem cells [10,67]. *In vivo*, these cells portray characteristics of NSCs. Gain-of-function studies forcing expression of *SOX* family genes including *SOX2*, *SOX1* and *SOX3*, maintained self-renewal and prevented neuronal differentiation [11,68]. Furthermore, inactivation of *SOX2* in loss-of-function experiments triggered a complete loss of GFAP/nestin positive NSCs and also reduced cell proliferation, whilst the presence of apoptotic markers increased [69]. Interestingly, the presence of OCT4, NANOG and other pluripotency TFs in NSCs as well as the normal brain has yet to be established. *SOX2* expression for normal stem cell function in the brain is dose-dependant. Mutations and deficiency in *SOX2* expression can underline several neurological diseases including hippocampal and motor abnormalities as well as epilepsy [70,71]. Overexpression of *SOX2* can lead to generation of cancer stem cells (CSCs) within the brain [7]. Therefore, understanding the normal function of pluripotency genes in NSCs and the biology of the stem cell niche can help in discerning mechanisms of brain repair and give insight into neurodegenerative diseases and brain cancer.

## 4. Aberrant Gene Expression and Tumourigenesis in the Breast and Brain

**Figure 2:**
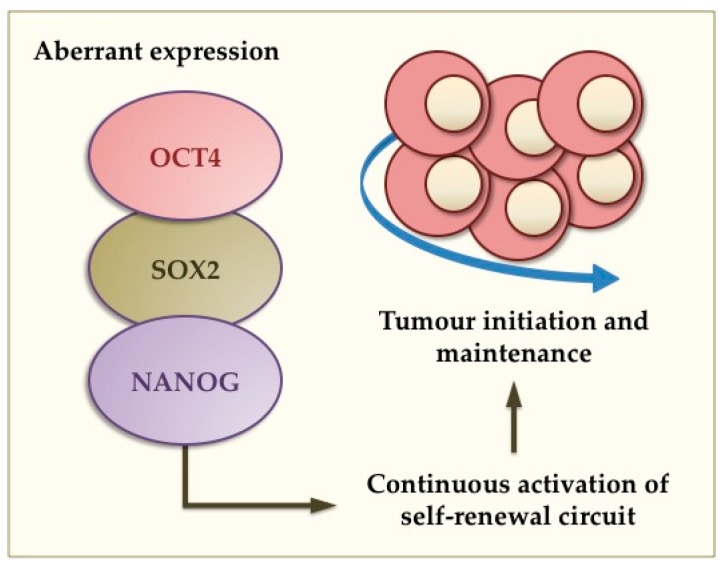
Proposed function of pluripotency TFs in brain and breast malignancies. The cancer stem cell (CSC) theory implicates that CSCs can express pluripotency genes such as *OCT4*, *SOX2* and *NANOG*. Aberrant expression of such genes causes continuous activation of the self-renewal circuit leading to oncogenic transformation, tumour initiation and maintenance.

In addition to their role in normal stem cell function, aberrant expression of pluripotency TFs has been strongly associated with cancer development. Solid tumours, such as those of the brain and breast, harbour a subset of cancer cells that have the ability to initiate and maintain tumourigenesis as well as resist conventional anti-cancer therapies [7,8,72,73,74]. Similar to normal stem cells, CSCs possess the ability to give rise into highly proliferative cells as well as more differentiated cancer cells representing several lineages that constitute the bulk of these heterogeneous tumours [8,72,75]. According to the CSCs hypothesis, normal somatic stem cells can undergo oncogenic mutations giving rise to stem-like cancer cells (Figure 2). Previous studies in brain and breast tumours have supported this as CSCs derived from these tumours are comparable to neural and mammary stem cells, respectively [75,76,77,78]. The CSC theory also implements that tumours have a hierarchical structure in which quiescent stem-like cells are favoured [79,80] as most anti-cancer therapies target highly proliferative cells. Therefore, anti-cancer therapies may enrich for the CSC population [78,81,82], which has the ability to turn into more proliferative cells and thus be responsible for tumour heterogeneity, treatment failure, tumour recurrence, and poor clinical outcomes.

CSCs have indeed been identified within brain and breast tumours, and overexpression of pluripotency TFs has been well documented in both of these tumour types [7,74]. In this context, they act as oncogenes. Human gliomas have shown expression of *SOX2*, *OCT4* and *NANOG*, postulating the CSC theory and mutagenic transformation from normal NSCs [7,83]. Aberrant upregulation of these oncogenes, and in particular SOX2, gives rise to a type of CSCs known as glioma stem cells (GSCs), which are able to turn into highly proliferative cells and show multilineage potential [7,79,83]. Expression studies of *OCT4*, *SOX2* and *NANOG* have also established a positive correlation with tumour grade, thus an association with poor prognosis [7,84,85]. Western blot results, mRNA levels and immunohistochemical data of several human glioma specimens demonstrated greater expression of *SOX2*, *OCT4* and *NANOG* in higher-grade gliomas than lower grade tumours [7]. Immunohistochemical analysis also showed strong nuclei localisation of these TFs confirming their functionality [7,85]. In addition, *in vitro* studies of glioma cell lines in conditions that promote stemness and tumoursphere formation increased expression of *SOX2*, *OCT4* and *NANOG* [73,86,87]. Tumourspheres also exhibited proliferative and tumour-initiating capabilities once transplanted into mice [87].

Of these three TFs, SOX2 appears to be a key player in both the normal brain and brain tumours. The importance of SOX2 in gliomas has also been demonstrated *in vivo* using transplantation of high-grade oligodendroglioma cells into immunodeficient mice after *SOX2* knockdown. These SOX2-depleted cells allowed mice to remain tumour-free, whereas controls formed lethal tumours [88]. Knockdown of SOX2 in GSCs of human glioblastoma, a grade IV glioma and most common primary brain tumour, ceased cell proliferation and tumourigenicity in immunodeficient mice [89]. These findings emphasise the essential role of SOX2 in the initiation, maintenance and recurrence of brain tumours. And although *OCT4* and *NANOG* have demonstrated a positive correlation with tumour grade, the oncogenic role of *OCT4* and *NANOG* and their importance in brain tumourigenesis has not been explored.

In addition to the brain, the oncogenic function of pluripotency genes has been demonstrated in the breast [9,74]. Mammary stem cells are thought to be susceptible to mutagenic transformation resulting in constitutive over-activation of the self-renewal circuit that enables aberrant proliferation of the deriving cancer cells [90]. Hence, breast tumours are also thought to harbour a population of CSCs, which are termed breast CSCs (BCSCs) [91]. Similar to gliomas, breast carcinomas overexpress *SOX2*, and this is associated with high rates of cell proliferation, tumourigenesis and pathological grade [92]. An extensive analysis of several sporadic node-negative breast tumour specimens showed that *SOX2* was preferentially expressed in basal-like and triple negative breast carcinomas [93], further implicating an association with poor prognosis and poorly differentiated phenotypic characteristics. In breast malignancies, in addition to *SOX2*, *OCT4* also appears to play a key role [9,94]. Normal human breast cell lines transduced with *OCT4* produced cells that portray characteristics of breast cancer cells, including tumour initiation and colonisation [95]. Transplantation of these cells into mice produced highly malignant tumours [95]. Clinical studies have also shown the importance of *OCT4* in breast oncogenesis, as overexpression is related to poorer post-operative survival rate, disease progression and metastasis [94].

NANOG has also been associated with poorer overall survival of breast cancer patients, suggesting a relationship between *NANOG* expression and tumour grade [96,97]. But, unlike SOX2, NANOG does not appear to be a primary driver of tumourigenesis in itself, and overexpression of *NANOG* alone does not trigger tumourigenesis [96]. However, aberrant co-expression of both *NANOG* and *Wnt-1* has demonstrated involvement of NANOG in promoting breast tumourigenesis and metastasis [96]. Thus, it has been suggested that OCT4, SOX2 and NANOG may act as prognostic markers for breast cancer patients. *In vivo* studies also demonstrate greater tumourigenic capabilities and higher expression of associated stem cell oncogenes in tumours with high *OCT4* expression [94]. Interestingly, recent studies showed that *OCT4* and *NANOG* are upregulated within the normal human lactating breast compared to the resting breast, but this upregulation is controlled under normal conditions, and has been speculated to serve important functions in the remodelling of the gland during pregnancy and lactation [9]. Imbalanced overexpression of these genes has been shown in breast tumours, especially those displaying lactating features [9].This further reinforces the CSC theory by defining a connection between the normal lactating breast and breast tumours, and the derivation of CSCs from normal stem cells that have undergone malignant transformation inducing oncogenic markers.

Collectively, the CSC theory postulates that brain and breast tumours consist of a population of CSCs that gain constitutive activation of pluripotency genes, particularly *SOX2*, *OCT4* and/or *NANOG*. The aberrant expression of these pluripotency TFs governs tumourigenesis and aids malignancy, therefore representing a promising therapeutic target to specifically eradicate CSCs.

## 5. Targeted Therapies via Silencers of Pluripotency Oncogenes

**Figure 3:**
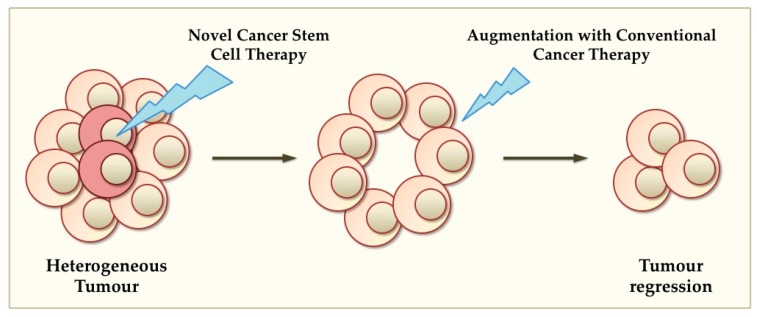
Potential of novel CSC-targeted therapies. According to the CSC theory, brain and breast tumours harbour a population of CSCs, which are refractory to current conventional therapy. Specifically targeting CSCs via silencing pluripotency oncogenes may eliminate CSCs and augment conventional therapies, resulting in tumour regression.

CSCs are quiescent and slowly cycling, which is thought to be a main characteristic that allows them to be refractory to current conventional chemotherapies and radiotherapy [98]. As described above, pluripotency TFs are involved in the control of tumourigenesis and cell proliferation in CSCs and their progeny, thus present novel therapeutic targets for these devastating diseases. Ideally, therapeutic strategies should specifically target CSCs through the aberrant expression of oncogenic TFs, and should augment current clinically used therapies (Figure 3).

Loss-of-function experiments and silencing studies of these TFs have further supported their role in tumourigenesis, conveying their potential as novel therapeutic targets. *SOX2* has been previously silenced in glioblastoma cells derived from patient tumour samples, resulting in a reduction in cell proliferation and tumourigenicity both *in vitro* and *in vivo* [89]*.* The observed anti-cancer effects were confirmed as a result of SOX2 loss [89]. Furthermore, use of SOX2 peptide vaccination in immunodeficient mice transplanted with high-grade oligodendroglioma cells delayed tumour development, increased survival rates, and the combination with chemotherapy drug temozolomide further doubled survival time compared to vehicle controls [88]. New technologies, such as engineered zinc finger-based artificial TFs, have been constructed to selectively silence *SOX2* gene expression in breast cancer cell lines, causing SOX2 mRNA downregulation and reducing cell proliferation and colony formation [99]. Mouse xenografts in the same study displayed significant reduction in tumour growth compared to wild type animals [99]. Similarly, small RNA interference technology against *NANOG* reduced cell proliferation, migration and colony formation of MCF7 and MDA-MB-231 breast cancer cells [100]. In this study, decreased expression of cyclin D1 and c-Myc suggested that knockdown of *NANOG* induced G0/G1 cell cycle arrest causing decreased cell proliferation [100]. Although studies have been performed on SOX2 targeted silencing, the potential of OCT4 as a target for brain and breast cancer is not well documented. However, ovarian cancer studies have shown that downregulation of *OCT4* via RNA interference promotes apoptosis and reduces cancer cell viability [101], indicating that OCT4-targeted interventions may also be promising in the breast and brain.

## 6. Conclusions

Our understanding of molecular pathways governing pluripotency in embryonic and adult stem cells has greatly improved in recent years. It has become apparent that pluripotency genes, in particular *OCT4*, *SOX2* and *NANOG* and their downstream targets, play a major role in maintaining the pluripotent state. Within the breast, pluripotency genes are likely crucial for normal mammary development during pregnancy and lactation, whilst in the brain they maintain the neural stem cell pool and control differentiation into functional brain cells. This highlights the therapeutic use of adult stem cells in regenerative medicine. Deregulation of pluripotency genes has been linked to inadequate mammary development and low milk supply during lactation. On the other hand, aberrant overexpression of pluripotency genes can give rise to aggressive cancer stem cells, which are present in solid brain and breast tumours, fuelling their maintenance and recurrence post-treatment. Hence, future studies should aim towards further examining the molecular pathways of OCT4, SOX2, and NANOG function in these and other organs, as well as their related downstream targets that systematically control normal tissue function and malignant transformation.
